# A Rare Case of Cutaneous Metastasis of Unresectable Rectal Adenocarcinoma

**DOI:** 10.7759/cureus.51550

**Published:** 2024-01-02

**Authors:** Sana Akhtar, Quratulain Khan, Anis Rehman, Muhammad W Khalid, Kashif Siddique

**Affiliations:** 1 Radiology, Shaukat Khanum Memorial Cancer Hospital and Research Center, Lahore, PAK

**Keywords:** surgery, chemoradiation, nape of neck, cutaneous metastases, rectal cancer

## Abstract

Colorectal cancer (CRC) is the third most common malignancy. Common metastatic sites for colorectal carcinoma are the lung and liver while cutaneous metastases are extremely rare. Skin metastasis may be an early manifestation of metastatic disease and represents a poor prognosis. Here we present a case of metachronous skin metastasis during chemoradiation treatment in a patient with locally advanced rectal cancer. A young boy aged 19 years presented to our hospital with radiological TNM staging of T3c N1 M0 with circumferential resection margin (CRM) involved. The treatment plan was defunctioning colostomy with neoadjuvant chemotherapy and radiotherapy with a later plan for surgery. Seven months later, there is a focal skin nodule in the nape of the neck. A core biopsy of this cutaneous nodule was done and proved metastatic. Surgery for the primary tumor and oligometastatic site was planned but due to extensive primary tumor, surgery was terminated and continues with chemotherapy and reassessment.

## Introduction

Colorectal cancer (CRC) is the third most frequently occurring cancer and the second leading cause of death, right after lung cancer. At diagnosis, almost 30% of people with CRC have a disease that has spread to other parts of the body. CRC commonly metastasizes to the liver, lungs, non-regional lymph nodes, and peritoneal cavity. Skin metastases of colorectal carcinoma are very rare with an incidence of 4% [1]. After cutaneous metastatic lesions, median survival is 18-20 months [2]. Lung cancer, breast cancer, and melanoma are the primary cancer types most frequently known to metastasize to the skin [3]. Different conditions that may appear similar to skin metastases include cysts, lipomas, granulomas, and neurofibromas. So, it is very important to do a biopsy of any suspicious cutaneous lesion [4]. In this case report, we will talk about the clinical presentation and imaging findings of cutaneous metastases during the treatment of advanced rectal cancer.

## Case presentation

**Figure 1:**
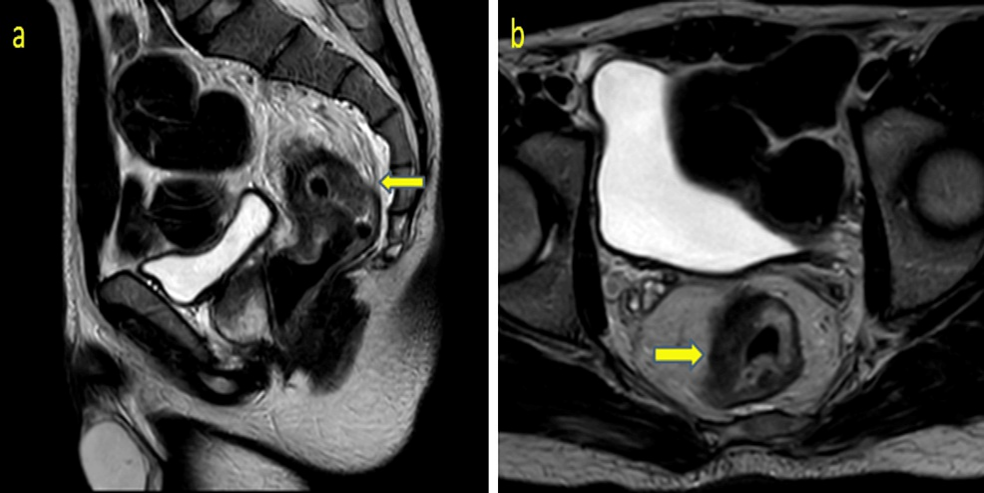
Sagittal (a) and axial (b) pelvis MRI scan T2 weighted images showed diffuse wall thickening of the rectum with mesorectal fat involvement (yellow arrows).

A 19-year-old male presented to our hospital with biopsy-proven poorly differentiated adenocarcinoma of the rectum with signet ring cell features. Colonoscopy was done which shows a circumferential stenosing tumor starting at 6 cm from the anal verge and extending proximally to 12 cm. The patient underwent an MRI pelvis (Figures 1a, 1b) that showed circumferential thickening of the mid and upper rectum with involved CRM and subcentimeter mesorectal lymph node, returning T2 intermediate signal and showing diffusion restriction. No enlarged pelvic sidewall lymph nodes were seen. No distant metastases were found on staging CT scan of chest and abdomen. 

**Figure 2:**
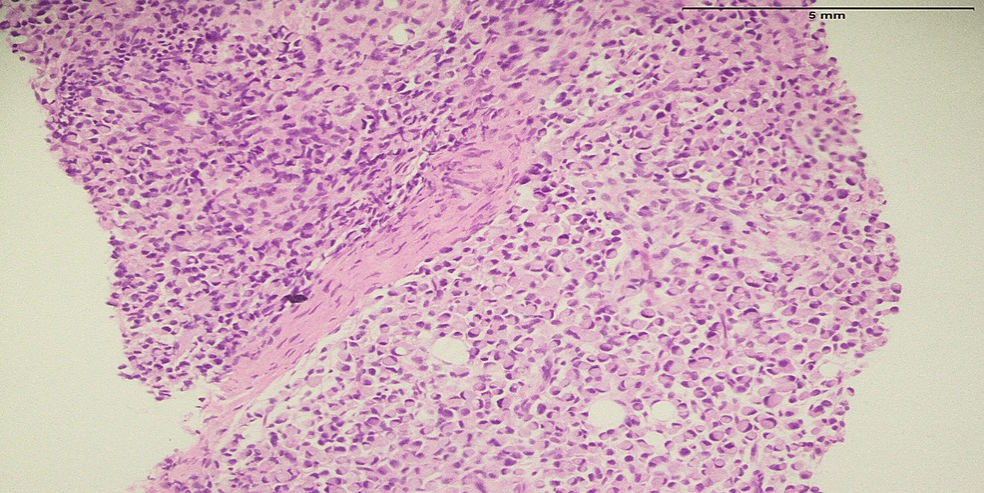
The section of the tissue core showed a malignant tumor composed of sheets of tumor cells with eccentric nuclei and vacuolated cytoplasm.

Defunctioning colostomy, neoadjuvant chemoXRT followed by assessment for surgery was planned in a multidisciplinary team. The patient underwent a defunctioning colostomy, chemotherapy, and radiotherapy. Seven months later, he presented with a painless swelling at the back of the neck. On physical examination, there was firm swelling over the nap of the neck on the left side with mild erythema, and the size of the swelling was approximately 3 cm. An ultrasound-guided trucut biopsy was performed on the lesion and the results revealed a malignant tumor with well-formed ductal structures that had invaded the dermis. The results supported the presence of metastatic cancer (Figure 2). The results of the immunohistochemistry analysis for mucin and cytokeratin 7 were positive.

**Figure 3:**
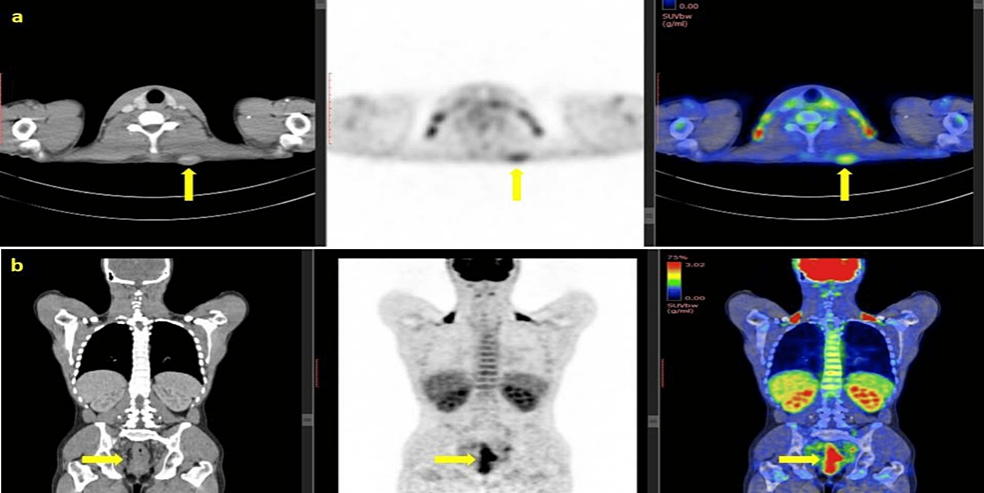
FDG-18 PET-CT scan demonstrated hypermetabolic lesion (a) at the nape of neck and (b) primary hypermetabolic rectal tumor (yellow arrows).

Subsequent fluorodeoxyglucose (FDG) positron emission tomography/computerized tomography (PET/CT) was done to identify additional sites of metastasis. A hypermetabolic primary rectal tumor and a left subcutaneous lesion at the nape of the neck were demonstrated by FDG-PET/CT. The rest of the scan was negative for any other site of metastases (Figures 3a, 3b).

## Discussion

Common metastatic sites for colorectal carcinoma are the liver, lungs, and bones. Cutaneous metastases are rare and occur in 4%-6.5% [2]. Cutaneous metastases are a poor prognostic factor [5] with an average survival ranging from three to 18 months [6]. The perineum and abdominal wall are the commonest sites for cutaneous metastases, while the head, neck, and limbs are rarely affected [7]. Mechanisms for cutaneous involvement are direct tumor extension, lymphatic or hematogenous dissemination, or implantation of tumor cells at the time of surgery [8]. Morphologically cutaneous metastases usually present as painless small nodules or ulcers with or without associated inflammatory reaction (cellulitis). The histological characteristics of metastatic lesions are usually more anaplastic and largely mimic the primary tumor. Microscopically, most skin metastases from colon cancer are nodular in appearance and develop in the dermis before spreading to the epidermis and subcutaneous tissue [9]. There were cases reported having isolated cutaneous metastases without visceral disease [10]. In a few numbers of rectal carcinoma patients, cutaneous lesions could occur before any other visceral metastases. Surgical resection, radiotherapy, and chemotherapy are the treatment options for cutaneous metastases. Even though treatment is not standardized for oligometastatic skin lesions of CRC, patients are managed to have limited metastases to other organs. They are started with upfront chemotherapy using 5 Flurouracil-based combination chemotherapy for three to six cycles and if there is no evidence of disease progression-definitive treatment to skin metastases with radiation/surgery is offered [11].

## Conclusions

There are not many case reports in the literature where cutaneous metastasis occurs during treatment for locally advanced cancer and is the initial symptom of metastatic disease. In our instance, the area of the metastatic site is highly tricky and may be mistaken for a sebaceous cyst or any benign skin lesion. Oncologists should remain vigilant for signs of skin involvement, even in cases of extended asymptomatic periods. Early identification of metastatic manifestations in the skin can significantly impact treatment strategies and prognosis in these cases.
